# Incidence of hypernatremia in dogs treated with multi-dose activated charcoal for acute toxicant ingestion: multi-center retrospective study (2018–2023)

**DOI:** 10.3389/fvets.2025.1584162

**Published:** 2025-05-12

**Authors:** Leah Gabriel, Rebecca A. L. Walton, Timothy Young, Jiazhang Cai, Jonathan P. Mochel, Katherine Peterson

**Affiliations:** ^1^VCA West Los Angeles, Los Angeles, CA, United States; ^2^Precision One Health Initiative, The University of Georgia, Athens, GA, United States; ^3^BluePearl, Duluth, MN, United States

**Keywords:** decontamination, electrolyte derangements, toxicity, sorbitol, hypernatremia

## Abstract

**Objective:**

To retrospectively evaluate the incidence of hypernatremia in dogs administered multi-dose activated charcoal (MDAC) for acute toxicant ingestion.

**Methods:**

Retrospective study between the years 2018–2023. Ninety-seven dogs evaluated by a university teaching hospital and private practice emergency hospital treated for acute toxicant ingestion with multi-dose of activated charcoal, with or without sorbitol.

**Results:**

Ninety-seven dogs were included. The median serum sodium concentration (Na) on presentation was 146.7 mEq/L (range 139–154.7 mEq/L), at 6–12 h 145.6 mEq/L (range 137–152.3 mEq/L), at 12–24 h 144 mEq/L (range 132.5–155) and at 24–48 h 144 mEq/L (range 134–150 mEq/L). Twenty-one dogs (21.6%) received 2 doses of AC, 37 dogs (38.1%) received 3 doses, 25 dogs (25.8%) received 4 doses, 3 dogs (3%) received 5 doses, and 11 dogs (11.3%) received 6 doses. There was no statistically significant difference in the type of toxicant ingested and changes in serum Na. No dog had a serum Na above 155 mEq/L. In dogs that received 2 doses of AC, there was no significant difference in serum Na at any time point. In dogs that received 3 total doses of AC there was a statistically significant decrease in serum Na at 12–24 and 24–48 h (*p* < 0.01). In dogs that had a total of 4 doses of AC, there was a statistically significant decrease in Na was noted at 12–24 h and 24–48 h (*p* < 0.01). For dogs that received 5 or 6 doses of AC, there was a significant decrease in serum Na at 6–12 h (*p* = 0.02). All dogs were hospitalized and 95 (98%) received intravenous fluids. The fluid rate and type were not significantly associated with changes in serum Na. Packed cell volume, total plasma protein, blood glucose and lactate on presentation were not significantly associated with change in serum Na at any time frame. All dogs survived to discharge.

**Conclusion:**

In this study, no dog receiving multi-dose activated charcoal developed hypernatremia and serum Na tended to decrease over time, which is unlikely to be clinically significant.

## 1 Introduction

Activated charcoal (AC) is utilized in the management of acute toxicant ingestion due to its porous structure and absorptive capacity (1). The addition of a cathartic, such as sorbitol (ACS), may hasten elimination of toxicants from the intestinal tract (2). Multi-dose activated charcoal (MDAC) theoretically benefits toxicant elimination by binding drugs that diffuse from circulation into the gastrointestinal lumen via enterohepatic or enteroenteric elimination (3, 4). Multi-dose AC, in comparison to single dose AC, has demonstrated significant reduction in elimination half-life in dogs with severe, experimental carprofen overdose (5).

The administration of MDAC rarely results in clinically significant side effects, however, potential complications include electrolyte derangements, including hypernatremia, gastrointestinal signs such as vomiting, diarrhea, or anorexia, or respiratory complications from aspiration (3). In humans, administration of MDAC is associated with a low incidence of clinically significant hypernatremia, with 6% of total patients having some degree of hypernatremia and 0.6% of patients developing serum sodium (Na) concentrations greater than 155 mEq/L. (6) Hypernatremia has been documented as a complication of single dose administration of AC in an experimental study in healthy dogs, however, a recent retrospective study evaluating the effect of single dose AC/ACS in acute toxicant ingestion in dogs found no dogs developed hypernatremia (7, 8). To our knowledge, no studies have evaluated the effects of MDAC on serum Na in dogs treated for acute toxicant ingestion. As MDAC is associated with a higher incidence of hypernatremia in people, compared to SDAC, the goal of this study was to evaluate the incidence and clinical relevance of hypernatremia after the administration MDAC in dogs with acute toxicant ingestion.

## 2 Materials and methods

### 2.1 Case selection

The computerized medical database of a university veterinary teaching hospital and a large, urban specialty hospital were searched for dogs administered MDAC with or without sorbitol for acute toxicant ingestion between February 2019 and August 2023. Search criteria included financial codes for “activated charcoal” and “activated charcoal with sorbitol.” Dogs were included in data analysis if MDAC/ACS was administered due to known toxicant ingestion and Na concentration were measured prior to administration and within a 6–48-h period following in the initial dose. Multi-dose AC/ACS was defined as 2 or more doses of AC/ACS. Dogs were excluded if they received a single dose AC, if AC was administered for reasons other than known toxicant ingestion, or if Na levels were not evaluated either prior to or following MDAC/ACS administration. Dogs with single dose AC/ACS administration were evaluated separately. The following data was collected: toxicant ingested, time from ingestion to presentation, induction and success of emesis, dose of AC/ACS administered, total number of doses and timeframe of multi-dose administration. Additionally, point-of-care (POC) bloodwork prior to administration of AC/ACS was recorded, including Na, chloride (Cl), packed cell volume (PCV), total plasma protein (TPP), blood glucose (BG) and lactate. Any repeat POC bloodwork at 6–12 h, 12–24 h, and at 24–48 h after initial dose of AC/ACS administration was also included where available. Additional treatment, including outpatient therapy or hospitalization was evaluated, along with the administration and rate of intravenous fluids and survival to discharge.

### 2.2 Statistical analysis

The aim of the study was to compare serum Na concentrations at presentation and at various time points following the administration of multi-dose activated charcoal. The comparison was made for each specific number of doses, with the number of doses treated as an ordinal variable. Due to the limited data for higher doses, dogs that received five and six doses of AC were grouped together. For each dosing group, Na concentrations at different time intervals (6–12 h, 12–24 h, and 24–48 h after the administration of MDAC) were compared to the Na concentration at presentation using the exact two-sample Kolmogorov–Smirnov test. Since each time point represented a broad time range (6, 12, and 24 h, respectively) and only three-time intervals were recorded, this hypothesis test was used to evaluate the effect of MDAC on Na concentration over time, rather than building a model to assess the relationship between time and Na levels.

The effect of several potentially relevant variables on Na changes at different time points using the Kruskal-Wallis rank sum test was also evaluated. These variables included the type of toxicant ingested, whether multiple toxicants were ingested, the time from ingestion to presentation, whether vomiting occurred before presentation, whether emesis was performed, and whether sorbitol was added to the first dose of activated charcoal. However, since some variables had multiple categories and Na concentrations were not measured for every dog at each time point, the Kruskal-Wallis rank sum test could not be performed in certain cases.

In this study, *p*-values less than 0.05 were considered statistically significant. All statistical analyses were conducted using R software (1), version 4.3.1. (11). ggplot2: Elegant Graphics for Data Analysis. Springer-Verlag New York. (ISBN 978–3–319-24277-4).

## 3 Results

A total of 472 dogs were identified through the medical records databases. Two hundred and seventy-nine dogs were excluded due to lack of point of care bloodwork including Na concentration following AC/ACS administration, incomplete records, or AC administration for noted reasons other than acute toxicant ingestion. Ninety-six dogs were excluded and evaluated as a separate population due to receiving single dose AC, therefore, 97 dogs were included in data collection. Thirty-seven were spayed females, 36 were male castrated, 13 were female intact and 11 were intact males. The median age was 3 years (range 0.3–13 years old). Median weight 18.6 kg (range 2–50 kg). The most commonly reported breed was Mixed Breed Dogs (16), Labrador Retrievers (9), Poodle (4), Chihuahua (4). Shih Tzu (4), and Maltese (4). Seven dogs presented less than 1 h post toxicant ingestion, 60 dogs presented 1–6 h post toxicant ingestion, nine dogs presented 6–12 h post toxicant ingestion, three dogs presented over 12 h post toxicant ingestion and 18 dogs had unknown timing of presentation compared to toxicant ingestion. The median serum Na concentration on presentation was 146.7 mEq/L (range 139–154.7 mEq/L), at 6–12 h 145.6 mEq/L (range 137–152.3 mEq/L), at 12–24 h 144 mEq/L (range 132.5–155) and at 24–48 h 144 mEq/L (range 134–150 mEq/L). Twenty-one dogs (21.6%) received 2 doses of AC, 37 dogs (38.1%) received 3 doses, 25 dogs (25.8%) received 4 doses, 3 dogs (3%) received 5 doses, and 11 dogs (11.3%) received 6 doses. The dosing frequency ranged from every 6–12 h in the majority of dogs but was unspecified in 5 dogs. The median dose of MDAC was 1 g/kg (range 0.85–2 g/kg).

In dogs that received 2 doses of MDAC, 11 dogs ingested an NSAID, 6 dogs ingested chocolate, 3 dogs ingested bromethalin and 1 dog ingested mushroom. There was no statistically significant difference in the type of toxicant ingested and changes in serum Na at any time point (Table 1). Twenty dogs ingested a single toxicant while 1 dog ingested multiple toxicants and the ingestion of multiple toxicants, compared to a single toxicant was not significantly associated with change in serum Na. Five dogs were noted to vomit prior to presentation and vomiting prior to presentation was not associated with change in serum Na. Emesis was performed and achieved in 14 dogs and was not significantly associated with changes in serum Na. All but 1 dog received ACS as the first dose, which was not associated with changes in serum Na.

**Table 1:** In dogs with acute toxicant ingestion that received 2 doses of activated charcoal, the association between variables and changes in serum sodium (Na) at 6–12 h, 12–24 h and 24–48 h.

Variable	Na at 6–12 h	Na at 12–24 h	Na at 24–48 h
Type of toxicant ingested	*p =* 0.25	*p =* 0.68	*p =* 0.56
Multiple toxicants ingested	*p =* 0.42		*p =* 0.44
Time from ingestion to presentation	*p =* 0.58	*p =* 0.37	*p =* 0.10
Vomiting prior to presentation	*p =* 0.18	*p =* 0.24	*P =* 0.44
Induction of emesis	*p =* 0.40	*p =* 0.09	*p =* 0.07
Addition of sorbitol to first dose of activated charcoal	*p =* 0.79		

Statistical significance set at *p* < 0.05.

In dogs that received 3 doses of MDAC, 21 dogs ingested an NSAID, 5 sago palm, 2 grapes/raisins, 2 chocolate and 1 of each of the following: cyclobenzamine, bromethalin, vitamin D, ephedrine, glucagon, coffee, mushroom. There was no association between type of toxicant ingestion and changes in serum Na (Table 2). Thirty-five dogs ingested a single toxicant while 2 dogs ingested multiple toxicants and there was no significant difference in multiple vs. single toxicant ingestion and changes in serum Na. Thirteen dogs vomited prior to presentation and 29 dogs had emesis successfully performed at presentation, both of which were not significantly associated with changes in serum Na. Thirty-three dogs received sorbitol with the first dose of AC, which was not associated with changes in serum Na.

**Table 2:** In dogs with acute toxicant ingestion that received 3 doses of activated charcoal, the association between variables and changes in serum sodium (Na) at 6–12 h, 12–24 h, and 24–48 h.

Variable	Na at 6–12 h	Na at 12–24 h	Na at 24–48 h
Type of toxicant ingested	*p =* 0.06	*p =* 0.21	*p =* 0.25
Multiple toxicants ingested	*p =* 0.80		*p =* 0.90
Time from ingestion to presentation	*P =* 0.56	*p =* 0.94	*p =* 0.83
Vomiting prior to presentation	*p =* 0.99	*p =* 0.33	*p =* 0.91
Induction of emesis	*p =* 0.49	*P =* 0.35	*p =* 0.43
Addition of sorbitol to first dose of activated charcoal	*p =* 0.71	*p =* 0.24	*p =* 0.76

Statistical significance set at *p* < 0.05.

In dogs that received 4 doses of MDAC, 17 dogs ingested an NSAID, 6 bromethalin and 1 of each of the following: vitamin D and mushroom. There was no association between type of toxicant ingestion and changes in serum Na (Table 3). Twenty-three dogs ingested a single toxicant while 2 dogs ingested multiple toxicants and there was no significant difference in multiple vs. single toxicant ingestion and changes in serum Na. Seven dogs vomited prior to presentation and 23 dogs had emesis successfully performed at presentation, both of which were not significantly associated with changes in serum Na. Twenty-three dogs received sorbitol with the first dose of AC, which was not associated with changes in serum Na.

**Table 3:** In dogs with acute toxicant that received 4 doses of activated charcoal, the association between variables and changes in serum sodium (Na) at 6–12 h, 12–24 h, and 24–48 h.

Variable	Na at 6–12 h	Na at 12–24 h	Na at 24–48 h
Type of toxicant ingested	*P =* 0.35	*P =* 0.35	*P =* 0.24
Multiple toxicants ingested	*p =* 0.37	*P =* 0.09	*p =* 0.51
Time from ingestion to presentation	*p =* 0.48	*p =* 0.88	*p =* 0.81
Vomiting prior to presentation	*P =* 0.35	*p =* 0.94	*P =* 0.79
Induction of emesis		*p =* 1.0	*p =* 0.87
Addition of sorbitol to first dose of activated charcoal	*P =* 0.48		

Statistical significance set at *p* < 0.05.

In dogs that received 5 or 6 doses of MDAC, 8 dogs ingested bromethalin, 5 dogs ingested an NSAID, and 1 dog ingested cholecalciferol. There was no association between type of toxicant ingestion and changes in serum Na (Table 4). All dogs ingested a single toxicant. Two dogs vomited prior to presentation and all dogs had emesis successfully performed at presentation. All but 1 dog received sorbitol with the first dose of AC, which was not associated with changes in serum Na.

**Table 4:** In dogs with acute toxicant ingestion that received 5 or 6 doses of activated charcoal, the association between variables and changes in serum sodium (Na) at 6–12 h, 12–24 h and 24–48 h.

Variable	Na at 6–12 h	Na at 12–24 h	Na at 24–48 h
Type of toxicant ingested		*P =* 1.0	*P =* 0.88
Time from ingestion to presentation	*p =* 0.32	*p =* 0.95	*p =* 0.38
Vomiting prior to presentation	*p =* 0.08	*p =* 0.48	*p =* 0.74
Addition of sorbitol to first dose of activated charcoal		*p =* 0.11	*p =* 0.83

Statistical significance set at *p* < 0.05.

All dogs had a serum Na performed at the time of presentation with a mean serum Na of 146.7 mEq/L (reference interval 143–155 mEq/L) (range 139–154.7 mEq/L) (Table 4). Forty-four dogs (45.5%) had serum Na concentration measured between 6 and 12 h post AC/ACS administration. Median serum Na concentration 6–12 h was 145.6 mEq/L (range 137–152.3 mEq/L). Seventy-seven dogs (79.4%) had serum Na concentration measured 12–24 h following the first AC/ACS administration with a median value of 144 mEq/L (range 132.5–153 mEq/L). Fifty-four dogs (56%) had serum Na concentration measured 24–48 h following initial AC/ACS dose with a median 144 mEq/L (range 134–150 mEq/L). No dog had a serum Na above 155 mEq/L at any time period.

In dogs that received 2 doses of AC, there was no significant difference in serum Na at the time of presentation and any time point (Table 4). In dogs that had 3 total doses of AC there was no difference in serum Na at 6–12 h (*p* = 0.35), however, a statistically significant, but not clinically significant, decrease in serum Na was noted at 12–24 and 24–48 h (*p* < 0.01 and *p* < 0.01). In dogs that had a total of 4 doses of AC, there was no significant difference in serum Na at 6–12 h (*p* = 0.16), but a statistically significant decrease in Na was noted at 12–24 h and 24–48 h (*p* < 0.01). For dogs that received 5 or 6 doses of AC, there was a significant decrease in serum Na at 6–12 h (*p* = 0.02).

All dogs were hospitalized and 95 (98%) received intravenous fluids. All dogs had access to water and no dogs had NG tube water provided. The median length of hospitalization was 47 h (range 15–168 h). The median rate of intravenous fluids was 80 mL/kg/day (range 30–160 mL/kg/day), 88 dogs were maintained with lactated ringers, 5 dogs were managed with Normosol-R and 2 dogs were maintained on saline. The fluid rate and type were not significantly associated with changes in serum Na. Packed cell volume, TP, blood glucose and lactate on presentation were not significantly associated with change in serum Na at any time frame. All dogs survived to discharge.

## 4 Discussion

The present study found that MDAC, with and without sorbitol, was not associated with the development of hypernatremia, defined as a serum Na greater than 155 mEq/L, in any dogs treated for acute toxicant ingestion. Trends of serum Na included statistically significant, but not clinically relevant, decreases in serum Na in dogs over the first 48 h of hospitalization.

Multi-dose AC interrupts enterohepatic and enteroenteric circulation of drugs, resulting in reduced systemic drug absorption, which may avoid the need for additional, invasive therapies such as hemodialysis and hemoperfusion (4). Multi-dose AC evaluated in an experimental study in healthy dogs was noted to reduce the area under the curve in carprofen ingestion and also reduced the elimination half-life of carprofen when compared to single dose AC (5). While often safe, MDAC has been associated with potential complications. In a study of 878 people who received MDAC, 0.6% of people were noted to have clinically significantly pulmonary aspiration and 0.6% of people were noted to have developed hypernatremia, defined as serum Na levels greater than 155 mEq/L (8). In dogs, SDAC has recently been evaluated in dogs with acute toxicant ingestion and dogs were noted to have a statistically significant but unlikely clinically significant decrease in Na over time (7). Multidose AC in dogs has been associated with vomiting and diarrhea, with a significantly higher incidence of vomiting in dogs receiving MDAC, as compared to single dose AC and MDAC administration, in the form of AC granules, has also been reported to result in a gastrointestinal obstruction (9). However, MDAC and its associated with hypernatremia has not been previously evaluated.

Hypernatremia, as a result of free water loss in the intestinal tract, is a potential concern with MDAC in dogs, however, no previous studies have evaluated the impact of MDAC in dogs for acute toxicant ingestion. This present study evaluated the impact of MDAC on serum Na in dogs for acute toxicant ingestion. No dog in this study became hypernatremic, defined as a serum Na greater than 155 mEq/L. In dogs that received 3 or 4 doses of AC, there was a statistically significant but unlikely clinically significant decrease in serum Na at 12–24 and 24–48 h.

The majority of dogs (98%) were managed with intravenous fluids. Serum Na is regulated and affected by free water balance and osmolality in the blood with a small contribution by hydration and volume status of the patient. Replacement intravenous crystalloids, which were used on the patients in this study, are generally used for fluid volume resuscitation and rehydration but may provide some free water to the patient (9, 10). The impact of fluid therapy could not be evaluated in this study due to the limited number of patients that did not receive fluids, however, both fluid rate and type were not significantly associated with changes in serum Na throughout hospitalization. These dogs were additionally all allowed free access to water, which was not quantified, therefore, water intake may have also resulted in appropriate free water fluid balance and the lack of hypernatremia in this study.

The association between POC diagnostics and changes in serum Na were evaluated in this study. Point-of-care diagnostics are often performed in acute toxicant ingestion and may be affected by presenting clinical status, including hydration or volume status. Dogs with acute toxicant ingestion that present with evidence of dehydration and hemoconcentration, which may be reflected by PCV and TPP values, may have had a higher incidence of developing hypernatremia following MDAC administration, however, this study found no association between POC diagnostics and changes in serum Na during hospitalization. Although PCV and TPP can be affected by variables other than dehydration, the mean PCV and TPP values were within reference range at the time of presentation in this study, indicating that dehydration was likely not clinically significant in dogs prior to MDAC administration.

There are several limitations due to the retrospective nature. The doses and frequency of MDAC were not standardized among patients. Additional therapies, other than intravenous fluids, were not evaluated and there was no information regarding water intake available for any dog, which may have affected Na concentrations. Finally, the timing of serum Na values was not standardized, which may have affected results.

In conclusion, hypernatremia did not occur in any dog receiving MDAC for acute toxicant ingestion. Multi-dose AC should be considered as part of the management in acute toxicant ingestion in toxicants that undergo enterohepatic circulation. Future directions include prospective, controlled evaluation of MDAC for acute toxicant ingestion in dogs with a more standardized dose and timing of Na rechecks.

## Data Availability

The original contributions presented in the study are included in the article/supplementary material, further inquiries can be directed to the corresponding author.
